# Anxiety, depression, perceived social support and quality of life in Malaysian breast cancer patients: a 1-year prospective study

**DOI:** 10.1186/s12955-015-0401-7

**Published:** 2015-12-30

**Authors:** Chong Guan Ng, Salina Mohamed, Mee Hoong See, Faizah Harun, Maznah Dahlui, Ahmad Hatim Sulaiman, Nor Zuraida Zainal, Nur Aishah Taib, on behalf of the MyBCC Study group

**Affiliations:** Department of Psychological Medicine, Faculty of Medicine, University Malaya, Lembah Pantai, 50603 Kuala Lumpur, Malaysia; Department of Psychiatry, Faculty of Medicine, Universiti Teknologi MARA, Kuala Lumpur, Malaysia; Department of Surgery, University of Malaya Medical Centre, Kuala Lumpur, Malaysia; Department of Social and Preventive Medicine, University of Malaya, Kuala Lumpur, Malaysia

**Keywords:** Breast cancer, Depression, Anxiety, Quality of life, Social support, Malaysia

## Abstract

**Background:**

Depression and anxiety are common psychiatric morbidity among breast cancer patient. There is a lack of study examining the correlation between depression, anxiety and quality of life (QoL) with perceived social support (PSS) among breast cancer patients. This study aims to study the level of depression, anxiety, QoL and PSS among Malaysian breast cancer women over a period of 12 months and their associations at baseline, 6 and 12 months.

**Methods:**

It is a 12 months prospective cohort study. Two hundred and twenty one female patients were included in the study. They were assessed at the time of diagnosis, 6 months and 12 month using Hospital Anxiety and Depression Scale (HADS), Quality-of-Life Questionnaire (QLQ–C30), Version 3.0 of the EORTC Study Group and Multidimensional Scale of Perceived Social Support (MSPSS). The information of age, ethnicity, types of treatment, and staging of cancer were collected.

**Results:**

The HADS anxiety and depression subscales scores of the subjects were relatively low. The level of anxiety reduced significantly at 6 and 12 months (Baseline – 6 months, *p* = 0.002; Baseline - 12 months, *p* < 0.001). There were no changes in the level of depression over the study period. The global status of QoL and MSPSS scores were relatively high. Correlation between the global status of QoL and MSPSS for the study subjects was positive (Spearman’s rho = 0.31–0.36). Global status of QoL and MSPSS scores were negatively correlated with anxiety and depression.

**Conclusion:**

Malaysian breast cancer women had relatively better QoL with lower level of anxiety and depression. Perceived social support was an important factor for better QoL and low level of psychological distress. It reflects the importance of attention on activities that enhance and maintain the social support system for breast cancer patients.

## Background

Breast cancer is the most common cancer diagnosed among Malaysian females [1]. Over the decades, the survival rate among breast cancer patients improves with early detection and advances in cancer treatment [2]. As such, the current focus in cancer treatment is not only about illness control but also the general wellbeing of the patients. Quality of life (QoL) is the measure of the patient’s perception of self-wellbeing. QoL encompasses several aspects of functioning such as psychological, physical, cognitive and social functioning [3]. Of the limited number of studies on QoL among breast cancer patients in Malaysia, a descriptive study involving 58 Malays and 15 Chinese women newly diagnosed with breast cancer found that the QoL was satisfactory based on the Malay version of the European Organization for Research and Treatment of Cancer Quality of Life Questionnaire (EORTC QLQ-C30). There was no difference in the QoL between the two ethnic groups [4]. Complementary and alternative medicine (CAM) is popular among cancer patients in Malaysia. In a cross-sectional study on QoL among breast cancer patients under chemotherapy, it found that the level of QoL was similar between CAM user and non-users [5]. In general, related studies showed that the general health status scores among breast cancer patients in Malaysia based on EORTC QLQ-C30 was about 60 to 65, which were considered satisfactory [4, 5].

Depression and anxiety are the two most common psychiatric comorbidities encountered in breast cancer patients [6, 7]. Breast cancer patient may experience depression and/or anxiety at any stage of their illness from pre-diagnosis to the terminal phase of the illness. Studies in the Western countries have shown that the prevalence of depression ranges from 1 % to 56 %, whereas the prevalence of depression from Asian studies is between 12.5 % and 31 % [8]. The difference in the prevalence rate of anxiety disorders depends greatly on the type of anxiety disorder being measured. A study by Dastan and Buzlu [9] reported that 35 % of their breast cancer patients had anxiety, while an Asian study reported a lower prevalence of 16 % [10]. According to Burgess et al. [11], it seemed that the prevalence of depression and anxiety among women with breast cancer declined from the first year of diagnosis to the fifth year after diagnosis from 48 % to 15 %. A study of depression and anxiety among Malaysian breast cancer patients found that the rate of anxiety was 31.7 % and depression was 22.0 %. Younger age, financial burden, and being single were the factors associated with anxiety or depression among the Malaysian breast cancer patients [12].

Social support is defined as any type of communication, in the forms of physical or psychological assistance, for someone to feel to have more self-control during difficult times [13]. Studies showed that the positive impact of social support does not derive from the people or institutions that form the social network. In contrast, they may be the cause of negative impact or stress for the individual. Instead, research has shown that it is the social support felt or perceived by the individual that produces the positive impact [14–16]. Perceived social support is negatively correlated with psychological distress and suicidal thought in cancer patients [17]. It is also associated with better orientation to health care services and adjustment to family relationship. Cancer patients with higher level of perceived social support have a better psychosocial adjustment to the illness [18]. Among the different domains, support from family and friends are shown to be associated with less breast cancer related distress [19]. Study of the association of perceived social support with QoL or distress among breast cancer patients in Malaysia is rare or non-existent. However, there is indication of association between emotional/informational support and better quality of life among the elderly in the rural community in Malaysia [20].

Cancer patients experience a fluctuating course of anxiety throughout the diagnosis and treatment phases [21, 22]. From the time of diagnosis, the cancer patients experience different types of mental distress and adaptation to the process of cancer treatment including investigation, waiting for results, planning for surgery, chemotherapy, hormonal therapy, radiotherapy and recovery [23, 24]. Interestingly, the result of a study by Kristin et al. in post-surgery breast cancer patients showed that QoL improved over the first 6 months. Anxiety remained unchanged after one year but was influenced by cancer diagnosis and treatment [25].

Little is known about the course of depression and anxiety in women with breast cancer in Malaysia. There is also no prospective study examining the changes in QoL of breast cancer patients and its relationship with depression, anxiety and perceived social support in Malaysia. In the current study, we aim to study the changes in QoL, depression and anxiety among Malaysian breast cancer patients from the time of diagnosis till one year of follow up visit. We also examined the association between the changes in QoL with depression, anxiety, and perceived social support in the subjects. .

## Methods

This study is part of a larger study, the Malaysian Breast Cancer Cohort (MyBCC), which is a prospective cohort study that aims to identify the association between genetics, lifestyle and nutrition on overall survival and quality of life of Malaysian Breast Cancer patients. For further detail of the cohort, please refer to the previous published report [26]. In the current study, we mainly focused on the natural course of anxiety depression and quality of life among the female patients at diagnosis till 12 months of follow up visit. The association between quality of life with anxiety, depression, and perceived social support for the subjects were also examined in this study.

Patients at the University of Malaya Medical Centre in Kuala Lumpur, Malaysia, who were diagnosed with breast cancer, were consecutively enrolled since 1 May 2011. Inclusion criteria were: (i) breast cancer that was confirmed by histological examination, (ii) able to complete the necessary interviews and questionnaires, and (iii) capable to understand the objective of the study and provide informed consent. Exclusion criteria were: (i) secondary breast cancer, (ii) having confusion or delirium, and (iii) male patients. The purpose and details of the study were explained to all potential subjects, and participants who gave written informed consent were enrolled. This study was approved by the Medical Ethic Committee, University of Malaya Medical Centre.

### Procedure and measures

All questionnaires and psychiatric measures were administered to patients by trained clinical research coordinators when patients were first diagnosed with breast cancer (baseline). The psychiatric measures were administered again at 6 months and 12 months follow-up visits thereafter. Information on age and ethnicity were obtained. Breast cancer staging was confirmed by the treating surgeon using the American Joint Committee on Cancer Staging System for breast cancer.

Anxiety and depression were assessed using the Malay Version of Hospital Anxiety and Depression Scale (HADS). HADS is the most frequently reported measure in cancer studies and shown to be the best performing measure for each trajectory stage of the disease. It is a self-administered questionnaire that screens anxiety (7 items) and depressive (7 items) symptoms. It has demonstrated good reliability. The anxiety (HADS-A) and depression (HADS-D) subscales are scored from 0 to 3 (four-point Likert scales), giving maximum scores of 21 for anxiety and depression respectively [27]. The Malay version of HADS has a good reliability and has been validated among the Malaysian population [28].

Quality of life was measured using the Quality of Life Questionnaire QLQ–C30, Version 3.0 of the EORTC Study Group on Quality of Life [29]. The QLQ-C30 is composed of both multi-item scales and single-item measures. These include a global health status/QoL scale and five functional scales evaluating physical functioning, role functioning, emotional functioning, cognitive functioning, and social functioning. The symptom scales and items were not included in this study. All the measures range in score from 0 to 100. Higher mean scores on these scales represent better functioning and QoL. The additional module QLQ–BR23 contains breast-cancer–specific scales. Similar with previous study, we only used three most relevant subscales: body image, arm symptoms, and breast symptoms [25]. Higher mean values on the arm- and breast-symptom scales indicate an increased extent of symptoms. Higher mean scores on the Body Image scale of the QLQ–BR23 represent better functioning [30].

Perceived social support was measured with Multidimensional Scale of Perceived Social Support (MSPSS). MSPSS is one of the self-administered scales. It is simple to be administered and scored. The scale consists of 12 questions and uses a 7-point Likert scale. The scoring ranges from “strongly disagree” to “strongly agree”. The scale has three subscales consisting of four questions to measure namely, family, friend, and significant other support. A high score represents high perceived social support [31]. It was translated into the Malay language and its psychometric properties have been established. The Malay version of MSPSS was shown to have high internal consistency of the total and each subscale, with excellent factorial validity [32].

### Statistical analysis

Descriptive statistics for age, ethnicity and staging of cancer was performed. Mean and standard deviation for all items in the QLQ-C30, HADS depression subscale and anxiety subscale scores, MSPSS total and each domain subscale scores were calculated for the baseline, 6 months and at the 12 months visits. The normality of the data was tested with Kolmogorov-Smirnov test. As the data was non-normally distributed, the QLQ-C30, MSPSS total and domain scores, HADS depression and anxiety subscale scores at 6 and 12 months were compared with baseline using the Wilcoxan test.

The correlation between QLQ-C30 with HADS and MSPSS at baseline, 6 months and 12 months were examined using Spearman test. Partial correlation between QLQ-C30 with HADS was further tested by controlling MSPSS scores. Likewise, the partial correlation between QLQ-C30 and MSPSS was tested by controlling the HADS scores. All the tests were two-tailed with alpha level of 0.05.

## Results

To the date of the current study in May 2015, 221 female subjects with breast cancer were recruited in MyBCC and had completed the 12 months follow up visit. The mean age of the subjects is 55.1 years old (SD = 11.5) with almost 50 % Chinese, followed by Malays (32.1 %) and Indians (17.2 %). Twenty six (11.8 %) had non-invasive breast cancer (stage 0), 60 (27.1 %) had Stage I, 83 (37.6 %) had Stage II, 44 (19.9 %) had Stage III and 8 (3.6 %) had Stage IV disease. Almost all (95.5 %) had surgery with more than half had chemotherapy (59.7 %) and radiotherapy (68.3 %). Only about one third had hormonal therapy (Table 1).

**Table 1:** Characteristics of the subjects (*N* = 221)

Characteristic	
Age, mean (sd)	55.13 (11.5)
Ethnicity, n (%)	
Malay	71 (32.1)
Chinese	108 (48.9)
Indian	38 (17.2)
Others	2 (0.9)
Staging	
0	26 (11.8)
I	60 (27.1)
II	83 (37.6)
III	44 (19.9)
IV	8 (3.6)
Surgery, n (%)	211 (95.5)
Chemotherapy, n (%)	132 (59.7)
Radiotherapy, n (%)	151 (68.3)
Hormonal therapy, n (%)	69 (31.2)

### Quality of life, perceived social support, depression and anxiety

Quality of life of the 221 subjects was measured using QLQ-C30 and QLQ-BR 23 at three time points; baseline, 6 months and 12 months as shown in Table 2. There is improvement in the global health status/QoL at 12 months as compared to baseline (Baseline – 12 months, *p* = 0.015) with no significant change at 6 month (Baseline – 6 months, *p* > 0.05). Among the five functioning scales, physical functioning shows significant improvement at 6 months (Baseline – 6 months, *p* = 0.001) and social functioning improves at 12 months (Baseline – 12 months, *p* = 0.03). There is significant improvement in emotional functioning at both 6 and 12 months (Baseline – 6 months, *p* = 0.002; Baseline - 12 months, *p* < 0.001). There are no significant changes in other two functioning scales, namely, role and cognitive.

**Table 2:** Quality of life, perceived social support, anxiety and depression among the study subjects (*N* = 221)

	Baseline mean (sd)	6 months mean (sd)	1 year mean (sd)	Baseline – 6 months *p* value^a^	Baseline – 12 months *p* value^a^
QLQ-C30					
Global health status/QoL	69.83 (17.23)	70.56 (16.61)	72.48 (15.68)	0.557	0.015
Functional scales					
Physical functioning	91.58 (13.87)	87.77 (15.02)	72.48 (15.68)	0.001	0.236
Role functioning	93.21 (14.18)	90.11 (19.03)	91.59 (15.65)	0.066	0.280
Emotional functioning	78.17 (20.44)	83.70 (21.53)	86.89 (16.99)	0.002	0.000
Cognitive functioning	89.44 (15.70)	86.26 (18.47)	89.44 (15.70)	0.060	1.000
Social functioning	91.18 (18.64)	92.49 (15.72)	94.47 (12.98)	0.448	0.029
QLQ-BR 23					
Functional scales					
Body image	92.61 (16.27)	91.39 (17.39)	94.85 (13.21)	0.575	0.151
Symptoms scales					
Breast symptoms	10.06 (14.39)	9.80 (14.53)	9.09 (12.92)	0.940	0.579
Arms symptoms	10.56 (18.30)	11.48 (18.09)	10.96 (16.70)	0.396	0.593
HADS					
Anxiety subscale	5.42 (3.68)	4.45 (3.39)	3.75 (3.11)	0.002	0.000
Depression subscale	3.90 (3.44)	3.91 (3.31)	3.60 (3.07)	0.932	0.428
MSPSS					
Significant others	24.53 (3.35)	24.10 (4.19)	23.95 (3.71)	0.510	0.104
Family	24.90 (3.10)	24.37 (3.93)	24.14 (3.34)	0.297	0.012
Friends	22.08 (5.37)	22.23 (4.91)	22.37 (4.76)	0.965	0.797
Total	71.50 (10.37)	70.70 (11.49)	70.46 (10.54)	0.666	0.404

QLQ–C30 = Quality-of-Life Questionnaire (QLQ–C30), Version 3.0 of the EORTC Study Group on Quality of Life; QLQ–BR23 Questionnaire = a breast cancer-specific module for the QLQ; HADS = Hospital Anxiety and Depression Scale, sd = standard deviation; ^a^Wilcoxan test

The mean scores of perceived social support of the subjects are relatively high. The mean score for the total scale is 71.50 (SD = 10.37), significant others subscale is 24.53 (SD = 3.35), family subscale is 24.90 (SD = 3.10) and friends subscale is 22.08 (SD = 5.37). There are no significant changes for the scores of the perceived social support from baseline at 6 months or 12 months.

Anxiety and depression were measured with HADS and reported at three time points. Depression is relatively low and does not change significantly at both 6 months and 12 months’ time point (Baseline – 6 months, *p* = 0.932; Baseline - 12 months, *p* = 0.428). There is significant reduction in anxiety at 6 and 12 months as compared to baseline (Baseline – 6 months, *p* = 0.002; Baseline - 12 months, *p* < 0.001) (Table 2).

### Correlation between quality of life with perceived social support, anxiety and depression

#### At baseline

The correlation between global health status, all functioning (physical, role, emotional, cognitive and social functioning) and body image with anxiety or depression is negative (*r* = −0.22 to −0.50). The correlations remained significant in partial correlation analysis after controlling for perceived social support scores. In contrast, breast and arm symptoms based on QLQ-BR23 are positively correlated with anxiety and depression. The correlation is also significant after controlling for the perceived social support scores (Table 3).

**Table 3:** Correlation between anxiety, depression and perceived social support with quality of life (*N* = 221)

Time point: Baseline												
	HADS				MSPSS							
	Anxiety subscale		Depression subscale		Significant other		Family		Friends		Total	
	*R*	*pr* ^*1*^	*r*	*pr* ^*1*^	*r*	*pr* ^*2*^	*r*	*pr* ^*2*^	*r*	*pr* ^*2*^	*r*	*pr* ^*2*^
QLQ-C30												
Global health status/QoL	−0.28^**^	−0.29^**^	−0.22^**^	−0.21^**^	0.27^**^	0.29	0.26^**^	0.24^**^	0.28^**^	0.25^**^	0.31^**^	0.30^**^
HADS												
Anxiety subscale	-	-	-	-	−0.08	-	−0.074	-	−0.15^*^	-	−0.12	-
Depression subscale	-	-	-	-	−0.13^*^	-	−0.09	-	−0.19^*^	-	−0.17^*^	-
Time point: 6 months												
QLQ-C30												
Global health status/QoL	-.286^**^	−0.24^**^	−0.32^**^	−0.28	0.31^**^	0.24^**^	0.28^**^	0.21^**^	0.35^**^	0.27^**^	0.36^**^	0.27^**^
HADS												
Anxiety subscale	-	-	-	-	−0.13	-	−0.11	-	−0.19^*^	-	−0.16^*^	-
Depression subscale	-	-	-	-	−0.17^*^	-	−0.12	-	−0.20^*^	-	−018^*^	-
Time point: 12 months												
QLQ-C30												
Global health status/QoL	−0.38^**^	−0.31^**^	−0.38^**^	−0.27^**^	0.29^**^	0.23^**^	0.27^**^	0.17^*^	0.25^**^	0.17^*^	0.31^**^	0.22^**^
HADS												
Anxiety subscale	-	-	-	-	−0.32^**^	-	−0.35^**^	-	−0.30^**^	-	−0.35^**^	-
Depression subscale	-	-	-	-	−0.38^**^	-	−0.41^**^	-	−0.35^**^	-	−0.40^**^	-

HADS = Hospital Anxiety and Depression Scale, MSPSS = Multidimensional Scale of Perceived Social Support, QLQ–C30 = Quality-of-Life Questionnaire (QLQ–C30), Version 3.0 of the EORTC Study Group on Quality of Life; QLQ–BR23 Questionnaire = a breast cancer-specific module for the QLQ, *r* = Spearman correlation coefficient, *pr* ^*1*^ = partial correlation controlling for MSPSS scores, *pr* ^*2*^ = partial correlation controlling for HADS scores; **p < 0.01; *p < 0.05

Only global health status has statistically significant and positive correlation with the MSPSS total and domain subscales scores. The correlation between functioning (physical, role, emotional, cognitive and social functioning), body image, breast and arm symptoms with perceived social support as measured with MSPSS after controlling for anxiety and depression are not statistically significant (Table 3).

#### At 6 months

The correlation between global health status, all functioning (physical, role, emotional, cognitive and social functioning) and body image with anxiety or depression (*r* = −0.22 to −0.50) is negative. The correlations with depression were no more significant in partial correlation analysis after controlling for perceived social support scores. Breast symptoms based on QLQ-BR23 are positively correlated with anxiety and depression. The correlation with depression was also not significant after controlling for the perceived social support scores (Table 3).

Similar to the finding at baseline, only global health status has statistically significant and positive correlation with the MSPSS total and domain subscales scores. The correlations of functioning (physical, role, emotional, cognitive and social functioning), body image, breast and arm symptoms with perceived social support as measured with MSPSS are not statistically significant (Table 3).

#### At 12 months

Although there exist negative partial correlation between global health status, most functioning (except role functioning) and body image with anxiety or depression, the correlations are lower as compared to baseline (*pr* = −0.15 to −0.38). The correlations remained significant in partial correlation analysis after controlling for perceived social support scores. Arm but not breast symptoms based on QLQ-BR23 are positively correlated with anxiety and depression. The correlation is also significant after controlling for the perceived social support scores (*pr* = −0.15) (Table 3).

At baseline and 6 months, the correlation of global health status with the MSPSS total and domain subscales scores is positive and statistically significant. Emotional and social functioning is positively correlated with total MSPSS, family and significant others subscale scores after controlling for anxiety and depression (Table 3).

## Discussion

This study showed that Malaysian breast cancer patients had relatively low levels of depression and anxiety and high QoL at the time of diagnosis. There is no change in the level of depression at 6 months and 1 year. In contrast, the level of anxiety reduced at 6 months and 1 year. The global health status, functioning, image status, breast and arm symptoms are all correlated with anxiety and depression. The correlation with depression is insignificant at one year. The correlation between global health status and perceived social support is positive and statistically significant. The level of perceived social support remained high throughout the 12 months period of study.

Breast cancer patients are known to suffer from high level of psychological distress at the beginning of the illness [11]. This is related to the fear and uncertainties toward the illness and its treatment. The reported prevalence of distress in breast cancer patients varies depending on the study design, settings and assessment tools. On average, the prevalence of depression and anxiety among cancer patients ranged from 20 to 30 % [8, 11, 33]. In the current study, the level of depression and anxiety was found to be comparable with previous studies. The average scores of both HADS depression subscale and anxiety subscale for the subjects in this study were lower than the threshold score for HADS defined anxiety or depressive disorder [27]. A striking finding was that the level of anxiety and depression did not change throughout the 12 months of follow up. This finding is different from the previous study. In the study by Stafford et al., the course of anxiety and depression of the breast cancer and gynaecological cancer patients were measured using HADS - Anxiety Subscale and Centre for Epidemiological Studies Depression Scale (CES-D) at diagnosis and again every 8 weeks for 56 weeks [34]. The authors found that anxiety and depression symptoms were highest at diagnosis with significant improvements observed by 8 and 24 weeks, respectively and maintained thereafter. The authors argued that the depressive symptoms did not decrease significantly at the beginning of the cancer due to the inclusion of physical symptoms in the assessment tool like CES-D, which they frequently encountered during the early treatment stage of cancer like surgery and chemotherapy [34]. However, in the current study, we studied the depressive symptoms using the HADS depression subscale, which does not contain somatic symptoms in the assessment [27]. In other words, the current finding demonstrated the persistency of depression for 12 months after the diagnosis in Malaysian breast cancer patients. This is in concordance with the finding by Ferrandina et al. Their study examined the quality of life and psychological distress among early stage cervical cancer patients and found no changes in the depressive symptoms after 12 months. In contrast, the anxiety symptoms in the study by Ferrandina et al. decreased over time starting from 3 months after surgery [35]. The illness and treatment related sequelae were recognized as the possible risk factor for psychological distress. For instance, lymphedema, which is a common consequence of breast cancer, has a negative impact on patients’ body image, ability to carry on with daily activities and social interaction.

QoL has become one of the main outcome measures in cancer treatment [21, 22]. QoL is a multi-dimensional measure of psychological, physical, role, cognitive and social well-being [3]. It is shown to be closely related to the illness progress, cancer treatment, underlying psychological condition, coping strategies and social support [11, 21, 22, 25, 36]. The scores of global health status of the study subjects were slightly higher than the Western studies [25, 36]. In other words, the general QoL was better in the current study group which was comparable with the findings of other studies in Asia [37]. We also demonstrated that the global health status of the breast cancer subjects improved after 12 months together with social and emotional functioning. This was similar to the result of previous study by Kristin et al. [25]. The improvement in QoL was expected at 6 months to a year as most patients should have completed and recovered from initial surgery, hospitalization, chemotherapy or radiotherapy [25]. At this point of time, the patients were expected to have recovered from the initial emotional reaction. This was reflected in the result of the current study where emotional functioning improved at 6 and 12 months. Another interesting finding of the current study was the improvement of social functioning. This was never reported in other previous studies. In contrast, there was report about the reduction in social, emotion and cognitive functioning, which was still apparent at 12 months after cancer diagnosis especially in the younger subjects. The authors argued that older women have developed adequate strategies to cope with disease and impairment [38].

The improvement in functioning and QoL is related to the coping strategies. Spiritual coping is an important coping strategy in stressful situation such as cancer. Malaysia is a multi-ethnic country composed mainly of Malays, Chinese and Indians. The better level of QoL among the subjects with breast cancer could probably reflect that Malaysian female patients turned to spiritual coping to counter their psychological distress. Furthermore, the use of complementary and alternative medicines (CAM) is common among the Malays. One local study found that 64 % of the Malay women with breast cancer were CAM users and they believed in the power of prayer and used Malay traditional medicine to assist in healing the body’s inner strength, to cure cancer, and to reduce stress [39]. Religious coping was also shown to be positively correlated with lower risk of psychological distress in other Asian studies [40]. There were qualitative studies which showed that women of different cultural background with breast cancer did have different psycho-social- cultural concerns.

Many studies have shown that QoL of cancer patients was negatively correlated with depression and anxiety [21, 22, 41, 42]. This was also reflected in the result of the current study. The negative relationship between QoL with depression and anxiety was noticed at all three time points in the current study at baseline, 6 months and 12 months. Two previous Chinese studies reported a correlation between anxiety and depression with poorer quality of life in breast cancer patients under chemotherapy or even post therapy [21, 42]. A German study highlighted that anxiety and depression were associated not only with the psychosocial but also the somatic aspects of QoL of breast cancer patients [36]. An integrative review to explore the factors contributing to the QoL of African American breast cancer survivor was conducted by Mollica et al. [43]. The result showed that psychological domains were most highly represented in QoL domain in related studies. In a review of related literature, the findings demonstrated that depression diminishes quality of life, affects compliance with medical therapies and reduces survival [22]. It argued that depression impairs interpersonal relationships, occupational performance, stress and perceptions of health and physical symptoms. Therefore, depression has a detrimental impact on patients’ overall quality of life.

In the current study, we demonstrated the positive correlation between QoL and perceived social support. The higher level of perceived social support among the breast cancer subjects was associated with better QoL. This finding is similar to several previous studies [17–19]. In general, the level of perceived social support measured using MSPSS was relatively good among the subjects in the current study in comparison with the other studies [17, 29]. This could in another way explain the relatively better QoL among the Malaysian breast cancer patients in this study. The result of a Turkish study showed that perceived social support is lower for family and friends especially in the advanced stage [18]. This could be due to the higher expectation of the patients from family and friends, physical dependency and increased discomfort. The study also showed that low level of perceived social support is associated with higher suicidal ideation among the cancer patients. In another Turkish study of breast cancer patients, the result demonstrated that social support is a detrimental factor of adjustment to the illness [44]. Social support, particularly from the family, affects the adaptation to the illness process. Patients seem to be more hopeful with better social support. In another German study, social support was also shown to be strongly associated with QoL [36]. The authors believed that social support encompasses various aspects such as emotional which include caring and concern, instrumental to the provision of goods and services; and information assistance. Social support was assumed to be the mediator or buffering of the association between psychological distress and QoL in cancer patients (buffering hypothesis) [45]. Not only in cancer patient, but social support seem to be associated with better coping among women with high risk of hereditary breast cancer. A Dutch study showed that family communication, perceived social support from family and friends are important factors for long-term adaptation and reduced psychological distress among women with high risk of hereditary breast cancer [46].

In most studies, anxiety and depression were shown to be negatively correlated with perceived social support in cancer patients [17]. Social support buffer or mediate between psychological distress and QoL [45]. Interestingly, our result showed that this negative relationship was not significant in the beginning but becoming most significant after 12 months. This is similar with other reports about the decline in social support and increase in psychological distress as the disease progresses [17–19, 44]. It reflects the breakdown of contact in the respondents’ social circle and exhaustion of social support when the disease progresses. At the same time, there is increased expectation in social support due to physical pain and more suffering at the later stage of cancer. Studies have shown that social support gradually drifted away during advancement of the disease [17]. Social support, particularly from family, is an important factor in the adjustment to social environment, adaptation to the disease progress and domestic environment [18, 19, 44]. As such, social support is an important protective factor for the emotional and physical well-being of the cancer patients.

There were several limitations in the current study. Firstly, the study was conducted in a tertiary hospital setting located in the capital city of the country. The group of study subjects may not be representable to the general population in the country. Secondly, some potential associated factors of depression, anxiety and QoL such as type of anti-cancer treatment, religiosity, understanding and knowledge of the illness and its treatment were not measured in the study. Lastly, the observational period of a year may be insufficient to demonstrate the changes of psychological distress in breast cancer patients. We should take into account the long term impact of the illness and treatment on the psychological well-being of breast cancer patients. The changes in psychological suffering may require a longer period of monitoring and assessment.

## Conclusions

In conclusion, Malaysian breast cancer patients have relatively low levels of depression and anxiety associated with better QoL for the first 12 months after the diagnosis. The higher level of QoL among breast cancer women in Malaysia was also associated with the relatively high level of perceived social support. Cancer is becoming a major cause of morbidities and mortalities across the globe. With the advancement in cancer treatment, there is increased attention in improving the QoL among the cancer patients. The reported level of distress and QoL among breast cancer patients varies depending on the coping strategies and level of acceptance in concordance with local culture, belief and health care support. The findings in the current study reflect the importance of work in improving the caregiver system for breast cancer patients. Care giver support group, educational program, and other activities that will enhance the social support system are likely to benefit the care giver and indirectly improve the QoL among the breast cancer patients. Prevention or reduction of psychological suffering among the breast cancer patients such as depression and anxiety could be achieved by improving perceived social support.
